# Supplementary material for: miPIE: NGS-based Prediction of miRNA Using Integrated Evidence
Source: Sci Rep. 2019 Feb 7;9:1548. doi: 10.1038/s41598-018-38107-z (PMC6367335; doi:10.1038/s41598-018-38107-z)
Supplement: Supplementary file 1 — Supplemental Material [file 41598_2018_38107_MOESM1_ESM.doc]

# miPIE: NGS-based Prediction of miRNA Using Integrated Evidence

R.J. Peace, M. Sheikh Hassani, J.R. Green

**Supplementary Information**

| *mmu* | *hsa* |
| --- | --- |
| *dme* | *bta* |
| *gga* | *eca:* |
|  |  |

Supplemental fig 1. Generalization performance of the miPIE classification pipeline over six test species. MiRDeep method is shown for comparison. 10CV indicates 10-fold cross-validation over test dataset indicated in figure title. All other series represent performance on the hold-out test species where training set is from species indicated in legend; *all* indicates the combination of all datasets other than the test dataset.

*mmu hsa*


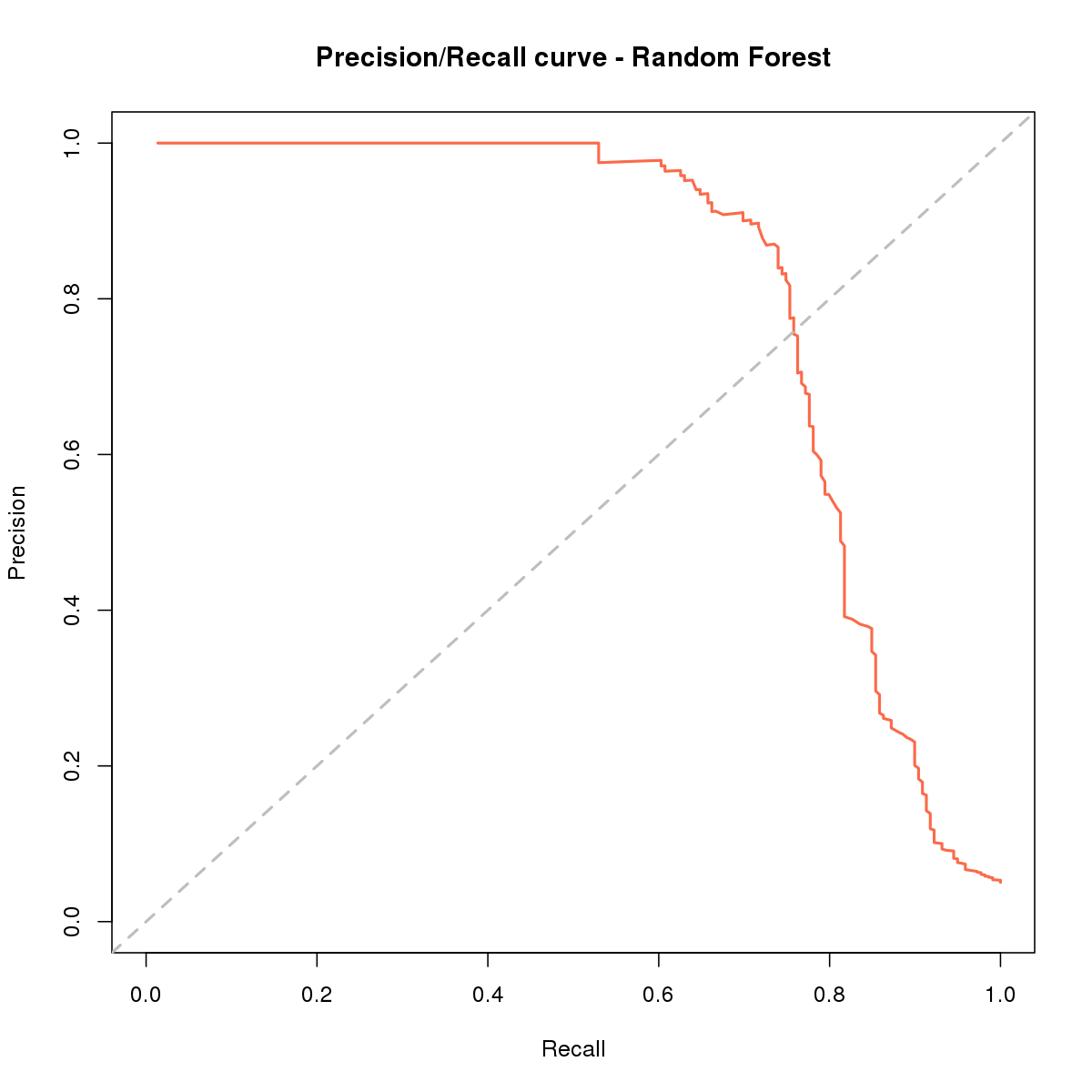

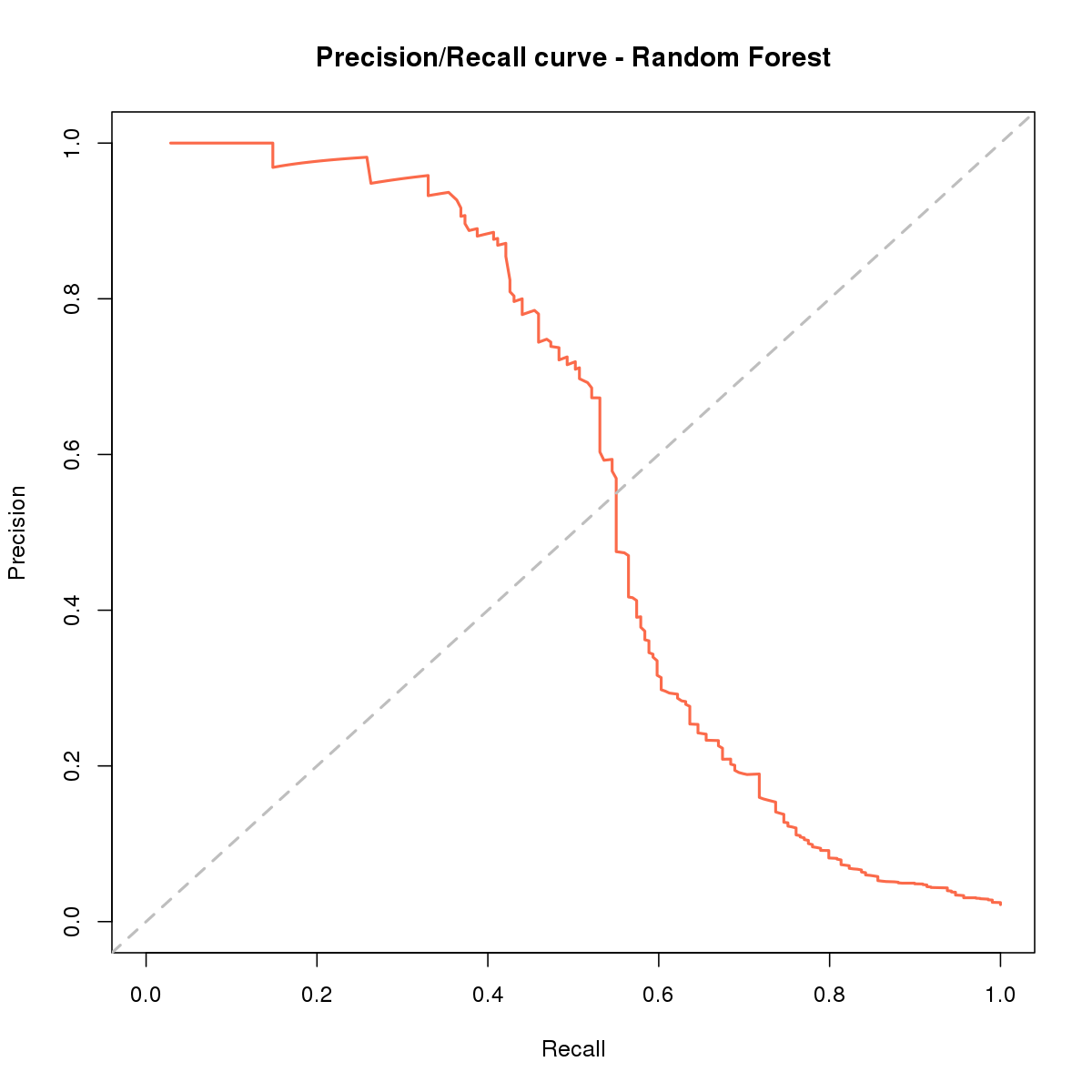


*dme bta*


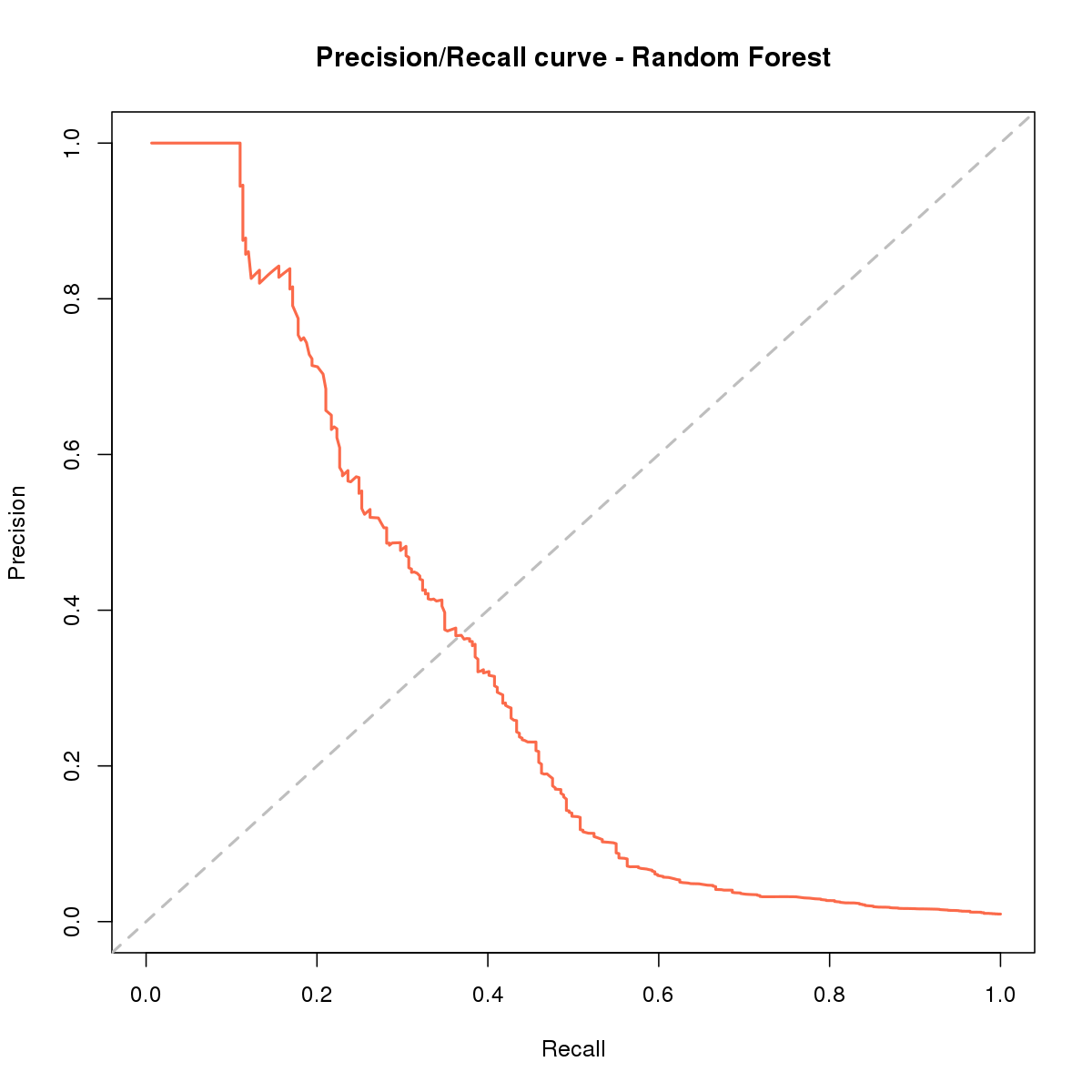

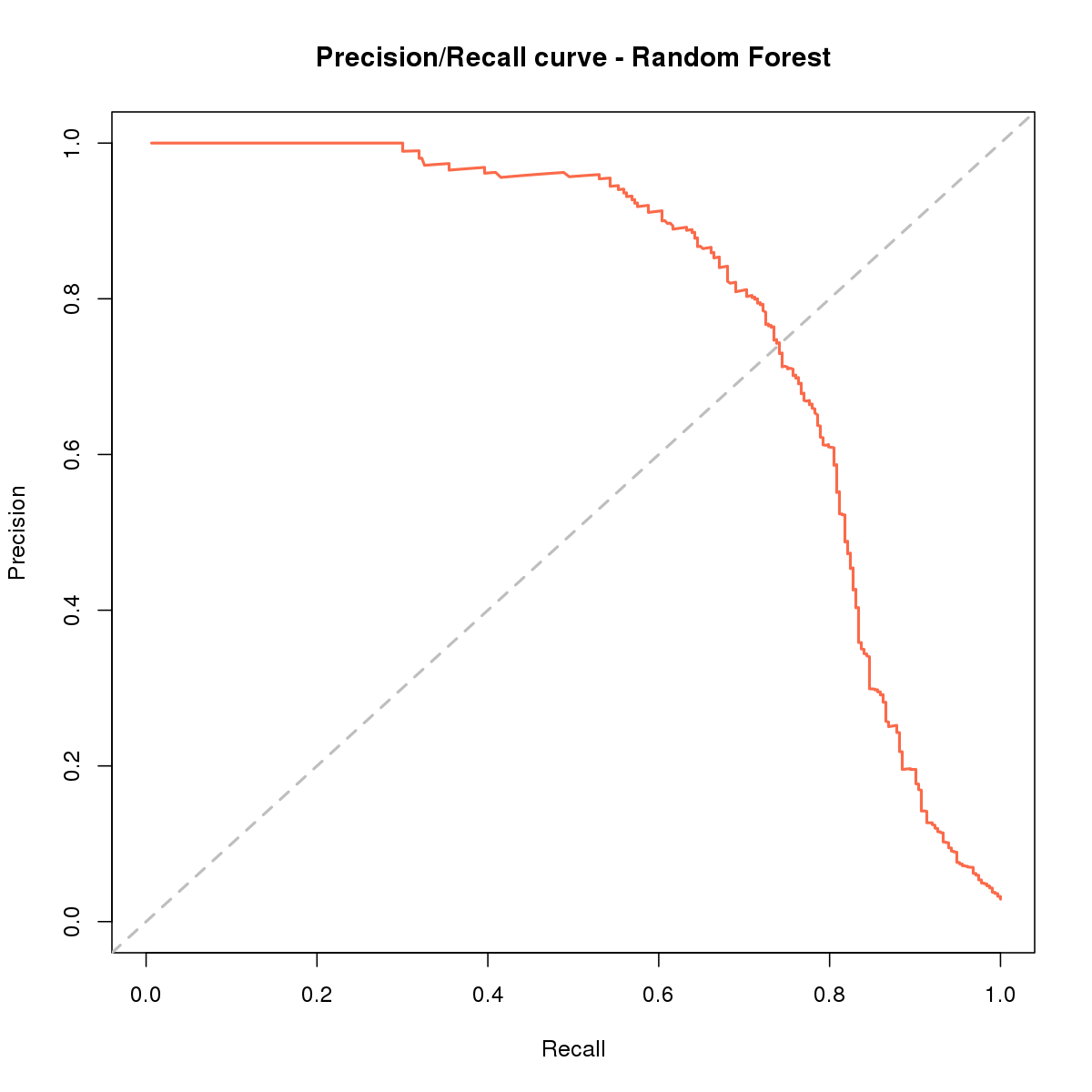


*gga eca*


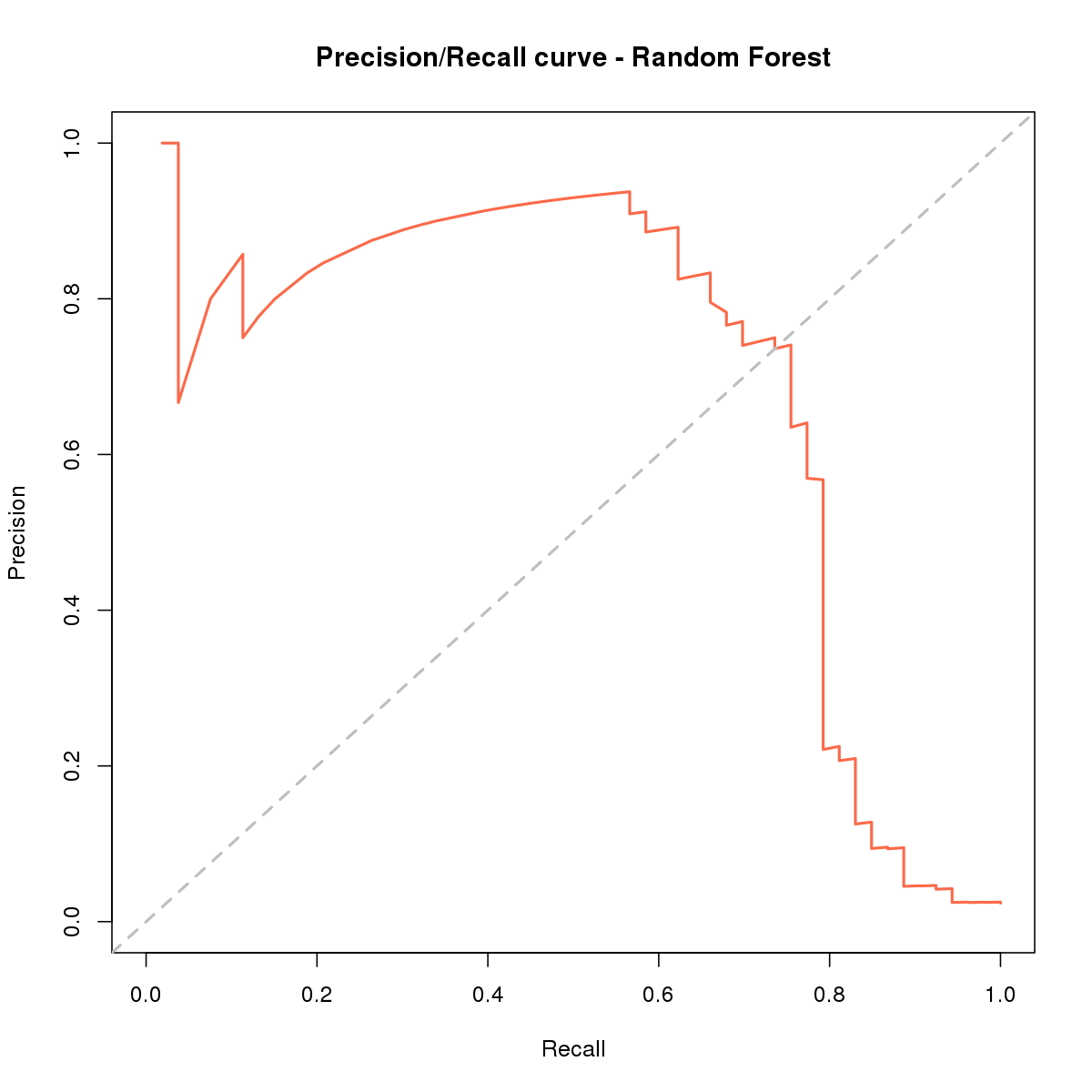

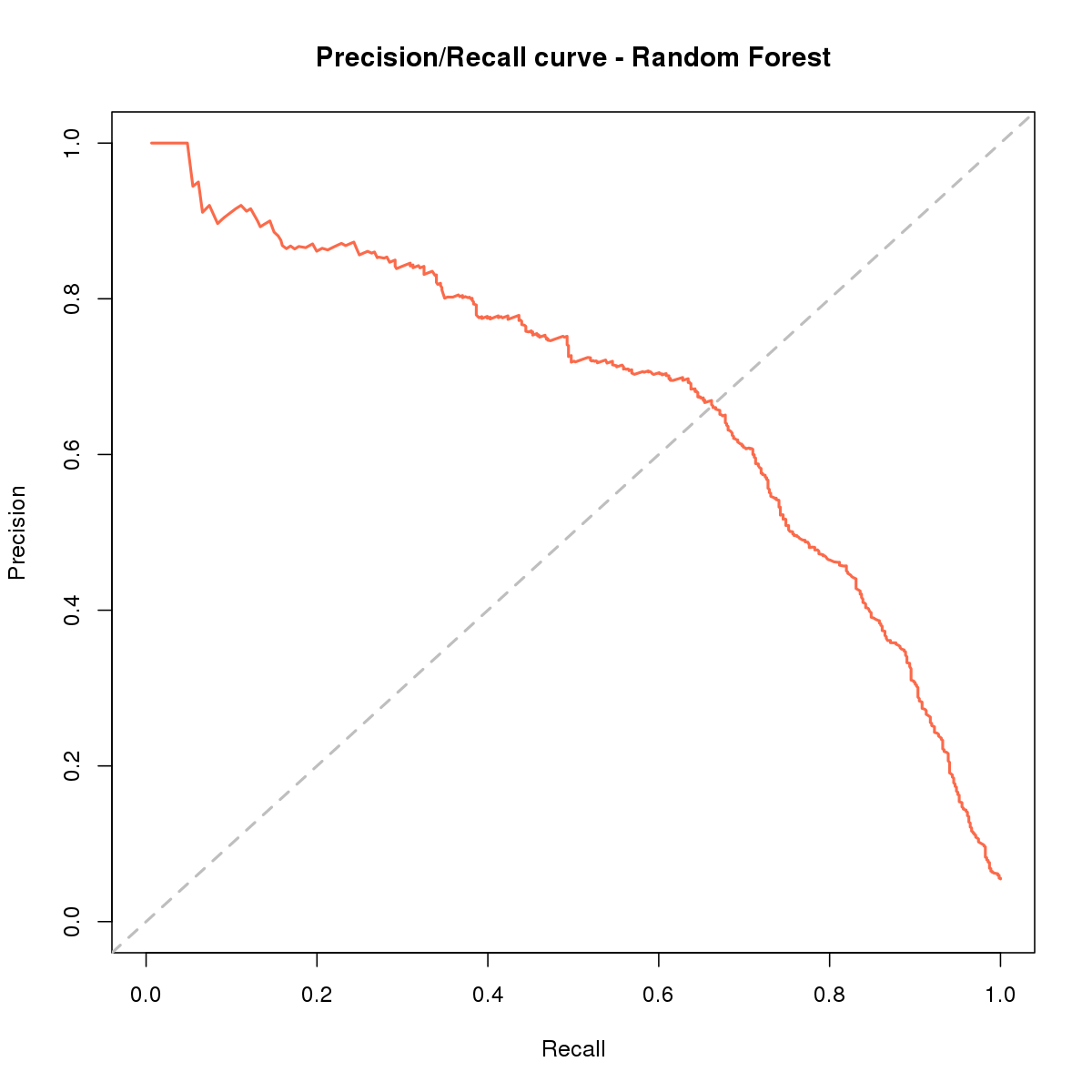


**Supplemental fig 2. Performance of mirnovo over the six data sets as given by the online tool. S**pecies-specific training models were used where possible (for mouse, human, fruit-fly and cow), while the universal animal model was used for the other two species (chicken and horse).

**Supplemental Table 1. Details of the expression- and sequence-based features selected by the correlation feature selection method for use in the miPIE prediction method**.

| **Feature** | **Description** |
| --- | --- |
| MFE3 | Ratio of normalized minimum free energy value and the number of loops in the pre-miRNA secondary structure. |
| dH | Enthalpy of the pre-miRNA secondary structure |
| Tm | Melting energy of the pre-miRNA secondary structure, and melting energy normalized by the loop length of the structure |
| Tm/loop |
| SC x zG | SC is a measurement of change in normalized structural stability when a pre-miRNA sequence is extended or reduced by equal amounts on the 5’ and 3’ arms. zG is the z-score of the normalized minimum free energy of the pre-miRNA secondary structure. dP is the number of paired bases normalized to the sequence length. |
| SC/(1 – dP) |
| Probpair2 | Sum of pairing probabilities of short nucleotide motifs |
| Probpair3 |
| Probpair7 |
| Probpair9 |
| Probpair19 |
| Probpair94 |
| C((. | Triplet motifs, representing a nucleotide identity and pairing of the 5’ neighbor, the nucleotide itself, and the 3’ neighbor of the nucleotide |
| T.(( |
| T..( |
| CG | Percentage of nucleotide dimers which contain the given nucleotide motif |
| GA |
| % pb mature | Percentage of paired bases in mature miRNA sequence |
| % reads mature | Percentage of reads within pre-miRNA sequence which align to mature and miRNA* regions |
| % reads miRNA* |

Supplemental Table 2. All novel miPIE predictions above 0.9 threshold (71):

Highlighting indicates sites predicted only by miPIE and not by miRDeep

| **Chromosomal location** | **miPIE score** | **Sequence** | **Secondary structure** |
| --- | --- | --- | --- |
| chrUn_NT_476229v1_39857 | 1 | GACCGGGGGGCGCGGCCCGCUUCAAGUAAUCCAGGAUAGGCUGUGCCCCCCCCGGCCUGUCCUCCGUUACUUGGGGUGGGACGCGCCCAGGCGGCCAUGUGUGGGGGGCGGG | ..(((..(((((((.(((((((((((((((..(((((((((((.(......))))))))))))..))))))))))))))).)))))))......(((....)))....))). |
| chrUn_NT_470375v1_39465 | 1 | CCCGGGGGGGCGCGGCCCGCUUCAAGUAAUCCAGGAUAGGCUGUGCCCCCCCGGCCUGUCCUCCGUUACUUGGGGUGGGACGCGGCCCAGGCGGCCAUGUGUGGGGGCGGGG | ((((..(((.((((.(((((((((((((((..(((((((((((........)))))))))))..))))))))))))))).)))).)))......(((....)))...)))). |
| chr7_34267 | 1 | CCGAAGAGGCUGGCGCUGGGUUCAAGUAAUCCAGGAUAGGCUGUGGUCUGGCAGUCAGCCUGUUCUAGGUUACUUGGCUCCGGAGCCCGCCCGACGCUUCGCCCUGGAGAUG | .(((((.(((.(((.(((((..(((((((((.(((((((((((..(.....)...))))))))))).)))))))))..))))).))).))).....)))))........... |
| chr2_17954 | 0.998 | UACAGAAGGCUGUCACCUGGUUCAAGUAAUCCAGGAUAGGCUGUAUCCAUUCCUGCUGGCCUAUUCUUGGUUACUUGCACUGGGAGGCAGCCGCAGUGCUGCAAAUGAGG | ..(((..(((((((.((..((.(((((((((.(((((((((((((........))).)))))))))).))))))))).))..)).)))))))......)))......... |
| chr6_33562 | 0.998 | AUUUGGCUCGUUGUUCCUUUUUCCUAUGCAUAUACUUCUUUGAGAGUUUGAUCUAAAGAGGCAUAGAGCAUGGGAAAAUGGGGCGACUGAGGUACUCCGCCAUUCAUUC | ...((((..((((((((.(((((((((((.(((.(((((((.(((......)))))))))).)))..))))))))))).)))))))).(((...))).))))....... |
| chr13_11956 | 0.996 | AGCAGCUGGGGGCUCCUCCAUGUCUCCCAGCCCAAGGUGCAGUGCUGCAUCUCUGGUCAAUUGGGAGUCUGAGAUGAAGCACUGUAGCUCGGGAAGGGAGGAACUGUGCCC | .((((.(((((....)))))..((((((..(((.((.(((((((((.((((((.((((......)).)).)))))).))))))))).)).)))..))))))..)))).... |
| chr2_21250 | 0.996 | CCGCGCCCGAGGAGGAUCCGGUCUCCUGAAGCAAAGUUCUGUGACACUCAGACUCUGGUUACGAUAGCAGUCAGUGCACUACAGAACUUUGUCUCCGGGGGCUGCGGCGGCG | ((((..((.....))...((((((((.((..((((((((((((.((((..(((((((.......))).))))))))...))))))))))))..)).))))))))..)))).. |
| chr19_14476 | 0.996 | GGCUGUACCAUCCUGUCGGAUAGCUUAUCAGACUGAUGUUGACUGUUGGAUCUCAUGGCAACAACAGUCGGUAGGCUGUCUGACAUUUUGGUAUCUCUCAUCUGACCGUU | ((..((((((...((((((((((((((((.(((((.(((((.((((........))))))))).)))))))))))))))))))))...))))))..))............ |
| chr1_2078 | 0.996 | CUGUGCUGCCAAUUGGCAUAAACCCGUAGAUCCGAUCUUGUGUUGAAAUGCACUGCACAAGCUCGCUUCUAUGGGUCUGUGUCAGUAUGGUGAUCUGGCAAAAGUUUA | CUGUGCUGCCAAUUGGCAUAAACCCGUAGAUCCGAUCUUGUGUUGAAAUGCACUGCACAAGCUCGCUUCUAUGGGUCUGUGUCAGUAUGGUGAUCUGGCAAAAGUUUA |
| chrUn_NT_467319v1_39026 | 0.994 | CCCAACCCUACUAUGUGUAUUUUGUGUCCCCCCCCCACUAUAUAAAACAGACCCCGUGCCCCCCCCGGGGUGAGGUAGUAGAUUGUAUAGUUGGGGGCUCACACGCCCGGCU | .((.....(((.....)))....((((...(((((.(((((((((.....(((...((((((....)))))).)))......))))))))).)))))...))))....)).. |
| chrUn_NT_467319v1_39025 | 0.994 | ACCCCGUGCCCCCCCCGGGGUGAGGUAGUAGAUUGUAUAGUUGGGGGCUCACACGCCCGGCUCGGAGAUAACUAUACAGUCUACUGUCUUCCCUGUGGGGGGUGUAUGGCAU | ...(((((((((((((((((.(((..(((((((((((((((((.((((......))))..........)))))))))))))))))..)))))))).)))))).))))))... |
| chr12_11207 | 0.992 | UACAUGCUUCUCUGUCAGAGUGAGGUAGUAGAUUGUAUAGUUGUAGGGUAGUUAUUUUACCCUGUUCAGGAGAUAACUAUACAAUCUAUUGCCUUCCCUGAGGAGUAAAACA | ....((((((((....((.(..(((((((((((((((((((((((((((((.....))))))).........))))))))))))))))))))))..)))))))))))..... |
| chr12_11208 | 0.992 | UAAUACUCGAGAAUGAGACUGUGGUUAGCUACUUCAGCAAAGAGCUUAAGUACAUGCUUCUCUGUCAGAGUGAGGUAGUAGAUUGUAUAGUUGUAGGGUAGUUAUUUUACCC | ................((((((((((.((((((((((((.(((((..........)).))).))).....))))))))).)))..)))))))...((((((.....)))))) |
| chr24_26206 | 0.99 | UUGACUGCAUGCAUCCAGGUUGAGGUAGUAGGUUGUAUAGUUUAGAAUUACACCAAGGGAGAUAACUGUACAACCUCCUAGCUUUCCUUGGGUCUUGCACAAAGCGGCGUG | ..(.((((.((((((((((..(((.(((.(((((((((((((.....(..(.....)..)...))))))))))))).))).)))..))))))...))))....)))))... |
| chr12_11210 | 0.99 | GAUGCCUGCACUGUGGGAUGAGGUAGUAGGUUGUAUAGUUUUAGGGUCAUACCCGCAACUGGGAGAUAACUAUACAAUCUACUGUCUUUCCUAAAGCAGCAGAAAAUCAAC | (((..((((.((.(((((.(((..((((((((((((((((.....(((...((((....)))).)))))))))))))))))))..)))))))).))..))))...)))... |
| chr26_26871 | 0.99 | UGUACUGCUCUGUGGAGGUGAGGUAGUAGGUUGUAUAGUUUGGUGGGAGGGAUUCUGUCCCAUUUCAGGUGAUAACUAUACAGUCUAUUGCCUUCCUUAAAGAGCAGCAAUA | ....(((((((...((((.((((((((((..(((((((((.(((((((.((...)).))))))).........)))))))))..))))))))))))))..)))))))..... |
| chr26_26872 | 0.988 | GUGCAAGAAAUCCUGCUCUUCCACAAAGCCAGGGAAUCACACCAGACCUCUGUACUGCUCUGUGGAGGUGAGGUAGUAGGUUGUAUAGUUUGGUGGGAGGGAUUCUGUCCCA | (..((.(((.(((....(((((((.((((...(.(((((((((..(((((..((......))..)))))..))).)).)))).)...)))).)))))))))))))))..).. |
| chr1_1805 | 0.988 | AGUCUGACUGUCCUUUGGGGUGAGGUAGUAGGUUGUAUAGUUUUAGGGUUAUGCCCUGCCUGUCAGAUAACUAUACAAUCUACUGUCUUUCCUGAAGUGGCUGUGAUAUCA | .(((..((.((((((..(((.(((..((((((((((((((((.(((((.....)))))..........))))))))))))))))..))))))..))).))).))))).... |
| chr1_NT_456233v1_random_16290 | 0.988 | GUCUGACUGUCCUUUGGGGUGAGGUAGUAGGUUGUAUAGUUUUAGGGUUAUGCCCUGCCUGUCAGAUAACUAUACAAUCUACUGUCUUUCCUGAAGUGGCUGUGAUAUCAU | (((..((.((((((..(((.(((..((((((((((((((((.(((((.....)))))..........))))))))))))))))..))))))..))).))).)))))..... |
| chr24_26204 | 0.988 | GACCCAUGCCUGUUGCCACAAACCCGUAGAUCCGAACUUGUGGUCAUAUUCCACACAAGCUUGUAUCUAUAGGUAUGUGUCUGUCUGGCAAGUGCACAAAGUGUUGUG | ......((((.(..(.((((.(((..(((((.(((.((((((...........)))))).))).)))))..))).)))).)...).))))....(((((....))))) |
| chr23_26080 | 0.988 | UUCGGGGCAGUCAUCGCUGCUGUAAACAUCCUUGACUGGAAGCUGUGAGGUGUCAGCGGGGGCUUUCAGUCGGAUGUUUACAGCUGCAGGCUGCUGCAGUCACCUGGGUG | ...(.(((((((...((.(((((((((((((..(((((((((((.(...((....)).).)))))))))))))))))))))))).)).))))))).)...(((....))) |
| chr4_32121 | 0.988 | AUCUUGGCAGUUCCCAGCUCUGAGAACUGAAUUCCAUGGACUGGUUUCAAUUCCAUGCGUUCAGUCCAUGGUAUUCAGUUCUCUAGCUUGGCUGCAUCAAACUUCACAACUG | ......((((..((.((((..(((((((((((.(((((((((((...((......))...))))))))))).))))))))))).)))).))))))................. |
| chr23_26082 | 0.988 | CCCUACCAUGCUGUAGCACGUGUAAACAUCCUACACUCUCAGCUGUGAACUCGAGGUGGCUGGGAGAGGAUUGUUUACGCCUUCUGCCAUGGAGUGAACAUCUGGUGAGCU | .....(((((..((((...(((((((((((((...((((((((..(.........)..)))))))))))).)))))))))...))))))))).((..((.....))..)). |
| chr3_29472 | 0.984 | GAGCUCUGAGUGACAGGUACUGUAAACAUCCUACACUCUCAGCUGUGGAAACUAAGAAAGCUGGGAGAAGGCUGUUUACUCUCCCUGCCUUGGAAAUCAUCUGGAGGGC | ((..((..((.(.((((....(((((((.(((...(((((((((..(....)......))))))))).))).)))))))....)))))))..))..))........... |
| chr1_7856 | 0.984 | UAAAUGUGGAAAACUUGUACCCCUUUCUACACAGGUUGGGAUCAGUUGCAAUGCUGUGCGUUUCUGUGGUAUUGCACUUGUCCCGGCCUGUUGAGGUUGGUGGGGAUAGAG | .............(((...((((..(((..((((((((((((.(((.(((((((..(........)..)))))))))).))))))))))))..))).....))))...))) |
| chr4_31646 | 0.984 | CCGAAGAUGCCUUGCGCUGGUUCCUCCGUGGGUGGGGAUUUGUUGCAUUACUUGUAGCUGUGUGUAGAGUAUUGCACUUGUCCCGGCCUGUGGAGGAAAGGAGGACUGGUC | ........(((.(.(.((..((((((((..(((.(((((..((.(((.(((((...((.....)).))))).)))))..))))).)))..)))))))).)).).)..))). |
| chr12_11115 | 0.984 | CCUCUUGCCUGAUUCCAGGCUGAGGUAGUAGUUUGUACAGUUUGAGGGUCUAUGAUACCACCCGGUACAGGAGAUAACUGUACAGGCCACUGCCUUGCCUGGGACAGAGCUC | ......(((((.((((((((.((((((((.((((((((((((.....((((.((.((((....))))))..)))))))))))))))).))))))))))))))))))).)).. |
| chr4_31648 | 0.982 | AAGAUGCCUUCCGGAGAGGUGCUGCUCACAGUCAGUUUUGCAGGUUUGCAUCCCAGCUUGCUAAAAUUGCUGUGCAAAUCCAUGCAAAACUGACUGUGGUGGUGGUGAAUAGC | .....(((((.....)))))((..(.((((((((((((((((((((((((...((((...........)))))))))).)).)))))))))))))))))..)).......... |
| chr1_7858 | 0.98 | UUUGUUGUUAAAUUGAACACUGUUCUCUGGUUAGUUUUGCAGGUUUGCAUCCAGCUGUAUGAUACUCUGCUGUGCAAAUCCAUGCAAAACUGACUGUGGCAGUGACAAGUCAG | .................(((((((...((((((((((((((((((((((..((((.(((...)))...)))))))))).)).)))))))))))))).)))))))......... |
| chr1_2082 | 0.978 | UCAUAUCAGACUUUUCCUAGUCCCUGAGACCCUAACUUGUGAGGUUUUGUAGCAACAAUCACAAGUCAGGCUCUUGGGACCUAGGCGGAGGGGAACCAGCAGCUUUGGAC | .....((...(((..(((((..((..(((.(((.((((((((.(((....))).....)))))))).))).)))..))..)))))..)))..)).((((.....)))).. |
| chr24_26208 | 0.976 | GCUCGUUGCGCCCCUCUCAAUCCCUGAGACCCUAACUUGUGAUGUUUAGCUUUUAAAUCCACGGGUUAGGCUCUUGGGAGCUGUGAGUUGUGCUUUGACAUCUUUAUUUU | ..(((..((((..(((.((.((((.((((.(((((((((((..((((((...)))))).))))))))))).))))))))..)).)))..))))..)))............ |
| chr2_23595 | 0.974 | GAUGUAUAGUAGUCUGUUGCUGUAAACAUCCCCGACUGGAAGCUGUAGCAGCUUGAGCUUUCAGUCAGAUGUUUGCUGCACCUGGCUAUACCUGGACAGCAUCAGGAGGAAAAG | ........((((((.(.(((.(((((((((...(((((((((((...........))))))))))).))))))))).))).).)))))).(((((......)))))........ |
| chrZ_43419 | 0.972 | GCCGGCGCAGAACGAGCCUGGGCUUGGAGCAGUGCUGAGAGGGCUUGGGGAGAGGAUUGUAGUGGAGCUCCAUCCCCAUUCCACUCCUAGCAGCUCUCUGGCCAUCCACC | ...(((.(((..(((((....)))))((((..(((((.(((((..((((((..(((..((......))))).))))))..)).))).))))))))).))))))....... |
| chr13_11953 | 0.972 | GCGGUCGCCGUGCUCUCAGGGUCCAGUUUUCCCAGGAAUCCCUUAGGCGCUACGUUGGGGAUUCCUGGAAAUACUGUUCUUGGGGCCACGGCUCUGCACCUGGAGACAGCCCG | ((((..((((((..(((((((..((((.((.((((((((((((..((((...)))))))))))))))).)).)))).)))))))..)))))).))))..(((....))).... |
| chr8_34624 | 0.97 | GAGGGGCCACGAUGACUGACAGGCUGCCCUGGCUCAGUUAUCACAGUGCUGAUGCUGUCUCUUGUAAAGGUACAGUACUGUGAUAACUGAAGGAUGGCAGCCAUCUGAGC | .....................(((((((.....((((((((((((((((((.((((..(....)....)))))))))))))))))))))).....)))))))........ |
| chrZ_40851 | 0.97 | GGCAUCAAACAAUAAGACUAUGAACUAUCCUUUUUCGGUUAUCAUGGUACCGGUGCUGUAUACGUGAAAGGUACAGUACUGUGAUAACUGAAGAAUGGUGGUGCCAUCACA | (((((((.......((........))..((((((((((((((((((((((..(((((............))))).)))))))))))))))))))).)))))))))...... |
| chr1_7862 | 0.968 | GUUCCAGUAGCUUUCUGCUUUGCAGUCUUCUGUUAGUUUUGCAUAGUUGCACUACAGGAAGAAUGUAGUUGUGCAAAUCUAUGCAAAACUGAUGGUGGCCUGUUAUAAUCUA | .....(((((....)))))..((((.((.(((((((((((((((((((((((((((.......))))...))))))..))))))))))))))))).)).))))......... |
| chr27_27144 | 0.968 | AUGCAUCUGUCUUCUAUAUGUACCCUGUAGAUCCGAAUUUGUGUAAAGGAAGUUGGGUCACAAAUUCGUAUCUAGGGGAAUAUGUAGUUGACACAAACACUACAGGUCACAA | .......((((..((((((((.((((.(((((.((((((((((...............)))))))))).))))))))).))))))))..))))................... |
| chr8_34310 | 0.964 | CGGAGAUCCUGCUCCGUCGCCCCAGUGUUCAGACUACCUGUUCAGGACAAUGCUGUUGUACAGUAGUCUGCACAUUGGUUAGACUGGGCAAGGGAAAGCAGCGACAUGGACU | ......((((((((.(((...(((((((.((((((((.(((.(((.......)))....))))))))))).)))))))...))).)))).)))).................. |
| chr17_13325 | 0.964 | GGCAGGGAGGGGAAGGACUAAUGAGCUGCUGAUAGCCGGAGAGCAGACCACAGAGGCUCCACUCCGUCUGCCCAGUGUUCAGACUACCUGUUCAGGACUACGAGAUUGUACA | (((..(((.(((.((......(((((.((((.(((.(((((.(.((.((.....)))).).))))).)))..)))))))))..)).))).)))..).))............. |
| chr17_13326 | 0.962 | CAGAGGCUCCACUCCGUCUGCCCAGUGUUCAGACUACCUGUUCAGGACUACGAGAUUGUACAGUAGUCUGCACAUUGGUUAGGCUGUGCUGGGAUACACCACACACUGCCAG | ....(((........(((((.(((((((.((((((((.(((.(((..........))).))))))))))).))))))).))))).(((.(((......))))))...))).. |
| chr3_29470 | 0.96 | UGCUGUUGACAGUGAGCGACUGUAAACAUCCUCGACUGGAAGCUGUGAAGCAGCAGAUGGGGCUUUCAGUCGGAUGUUUGCAGCUGCCAACUGCCACAGACGUCAAGAAA | ..((((.(.((((..(((.((((((((((((..(((((((((((.(............).))))))))))))))))))))))).)))..))))).))))........... |
| chr19_14784 | 0.96 | CCUCUGCCCGCUCCUGGCUGCCCGGCAGCAGUUCUUCAGUGGCAAGCUUUAUGUCCUUCUCUAGUAGCUAAAGCUGCCAGUUGAAGAACUGUUGAAUGUAGCCACGUUCAUC | .........(..(.(((((((....(((((((((((((((((((.((((((.((.((.....))..))))))))))))).))))))))))))))...))))))).)..)... |
| chr12_10416 | 0.96 | CUCUGCAGCGCCGGCACGGGCAACGGAAUCCCAAAAGCAGCUGUCCCCGCAGCGCCCAGCUGCCCUGGGAUUUCGUUACCCGCGCCUCCGCCUCAGCCGCCGCCGCUACGC | ....((.(((.((((.((((.((((((((((((...(((((((..(.......)..)))))))..)))))))))))).)))).((....))....)))).))).))..... |
| chr1_7859 | 0.958 | AUGACAGCUCUUGUAGCACUAAAGUGCUUAUAGUGCAGGUAGUGUUCACUAAUCUACUGCAUUAUAAGCACUUAAAGUACUGCUAGCUGUAGAACUACAUAUUCAGCAUGUU | ...((((((...((((.(((.(((((((((((((((((.((((....)))).....)))))))))))))))))..))).)))).))))))...................... |
| chr1_936 | 0.95 | CCGGGUGCGCCAGGCCUGGCUGAGGUAGUAGUUUGUGCUGUUGGUCGGGUUGUGACAUUGCCCGCUGUGGAGAUAACUGCGCAAGCUACUGCCUUGCUAGUGCUGGUGAUG | .......((((((..(((((.(((((((((((((((((.(((((.(((((.........)))))))........))).))))))))))))))))))))))..))))))... |
| chr2_23593 | 0.95 | UUAAGCUAACUUUUAGUUCCUGUAAACAUCCUACACUCAGCUAUAACAAGUGGUAGGGCUGGGGGGUGGAUGUUUACUUCAACUGACUUGGAAAGGCAGCUUUCCUGAAUGA | ............((((((...((((((((((.((.(((((((...((.....))..)))))))..))))))))))))...))))))...((((((....))))))....... |
| chrZ_41523 | 0.948 | CUGCACAGCUUUCUUUUAAUGUUGUGGCUGUUUGGGUUCCUGGCAUGAUGAUUUGUGAGUUAAGAUUAAAAUCACAUUGCCAGGGAUUACCACAUAGCCAUGACCGCA | ....................((..(((((((.(((((((((((((((.((((((...(((....))).)))))))).))))))))))..))).)))))))..)).... |
| chr33_30699 | 0.948 | GGGUGCCCACCCACCCUGGCAGCGCCCGGGCCGAGGUUCUGUCCUACACUCCGGCUGUGGCUAUGGGCAGUCAGUGCAUCACAGAACUUGGUCCCGGGAGCUCAGCAG | (((((......)))))..(((((.(((((((((((.((((((....((((..((((((........))))))))))....))))))))))).)))))).)))..)).. |
| chr2_18747 | 0.946 | GGGAGCAGUUCCACAGGAAUGUCCAGCUGGAAGGGGGGCCGUUACACUGUAAGAGAGUGAGUAGCAGGUCUCACAGUGAACCGGUCUCUUUUCCUGCUGUGUCAUGCC | ((((....))))...((.(((.(((((.(((((((((((((...((((((..((((.(........).))))))))))...))))))))))))).)))).).))).)) |
| chr7_34091 | 0.946 | CAUUAUGGGAUGGCCUGACUUUGAGCUGUUGAAUUCGGGGCCGUAACACUGUCUGAGAGGUUUAUAUUUCUCACAGUGAACCGGUCUCUUUUUCAGCUGCUUCCUGGC | ............(((.......((((.((((((...(((((((...((((((..(((((((....)))))))))))))...)))))))...)))))).))))...))) |
| chr20_24659 | 0.944 | CACCUUUGAGGGAGCGGCAGUUAAGACUUGUAGUGAUGUUUAGAUAAUGUAUUACAUGAACAUCACUUUAAGUCUGUGCUACUUCUCUCCUCAUUCUUGUCGGCGGGAAGG | ..((..((((((((.((.(((..(((((((.(((((((((((..(((....)))..))))))))))).)))))))..))).)).))).))))).(((((....))))).)) |
| chr4_32054 | 0.942 | UUCUCUGGACAUGACUGUAUCUCUGUGCUUUCAGCUUCUUUACAGUGCUGCCUUGUUGCGUUCAUGUCAAGCAGCAUUGUACAGGGCUAUGAAAGAACAGAGGCAUGCUCUU | ......(..((((......(((((((.(((((((((.((.(((((((((((.(((..(((....))))))))))))))))).)))))..)))))).)))))))))))..).. |
| chr13_11753 | 0.94 | GAAGAUGAAAAGAACAUCUGUCUUGCUGCCUUCGGCUUCUUUACAGUGCUGCCUUGUUGCAUAUGGAUCAAGCAGCAUUGUACAGGGCUAUGAAGGCACUGAGUCUUCUCU | ((((((...((((.(....)))))..(((((((((((.((.(((((((((((.(((.(.(....).).)))))))))))))).))))))..)))))))....))))))... |
| chr4_31644 | 0.94 | CACGGUAAGCUUUGUUUUGCUGUUGUCGGGUGGAUCACGAUGCAAUUUUGAUUAGUUUAGUAGGAGAAAAAUUGCACGGUAUCCAUCUGUAAACCGCAAGACCUUCAUCC | ...((((((....(((((((.(((..(((((((((..((.(((((((((..((..........))..)))))))))))..)))))))))..))).)))))))))).))). |
| chr28_28209 | 0.938 | CUCUGUGGUCUGGCUCUGUGUGGAAGACUAGUGAUUUUGUUGUUAUGAUUUAUAAAGGUGACAACAAAUCAUAGCCUGCCAUACAGCACAGAUCUUCACCCUUGCUGGUAGGAA | .(((..((((((...((((((((.((.(((.((((((.((((((((...........))))))))))))))))).)).))))))))..))))))...(((......))).))). |
| chrZ_45173 | 0.936 | GAUGUUGGUCUAGUUCUGUGUGGAAGACUAGUGAUUUUGUUGUUUUUAGAUAAUUAAAUUGACAACAAAUCACAGUCUGCCAUAUGGCACAGAUCAUGCCUCUACAGGACAAGU | ((.((((((((.((.((((((((.(((((.(((((((.(((((((((((....)))))..)))))))))))))))))).)))))))).)))))))).)).))............ |
| chr10_9089 | 0.936 | AGGACGGCCGCGGUGCCCUCUGGAAGACUAGUGAUUUUGUUGUUGUAUGGCUCAUCCCACCACAACAAGUCACAGUCUGCCUUAGGGCGCACGGCCCCGCCGGCGCUGCAGGAG | .((..(((((..(((((((..((.(((((.(((((((.(((((.((..((......)))).))))))))))))))))).))..))))))).))))))).((.((...)).)).. |
| chr2_23337 | 0.934 | UCUGCUUGCUCUGGUGGAGAUAUUGCACAUUACUAAGUUGCAUGUUGUCACGGCCUCAGUGCAAUUUAGUGUGUGCGAUACUUUCACAUGAGUGCAUGCACACGGGUAUGG | ..(((.(((.((.(((((((((((((((((.(((((((((((((..((....))..)).))))))))))))))))))))).)))).))).)).))).)))........... |
| chr2_19127 | 0.932 | GGGUUCUCACACACUCUGCAGCUUCCUGGGAUGGAGUUGUCCUUGUGCUGGACAGCAGAGAGGAGGCAAAGCAUACUGACACUCCACAGGCAGCCAGUGGAGAUGAAAUCAUUA | .((((.(((....(((..(.(((.((((...((((((.(((..((((((...(..(......)..)...))))))..))))))))))))).)))..)..))).))))))).... |
| chr1_7865 | 0.93 | GGUUGCGUCAGAGUAAUGUCAAAGUGCUUACAGUGCAGGUAGUGAUAUAUAGAACCUACUGCAGUGAAGGCACUUGUAGCAUUAUGUUGACAGCUGCCUCAGGAGAUCUUGC | (((.(((((((.(((((((..((((((((.((.(((((...((..........))...))))).)).))))))))...))))))).))))).)).))).((((....)))). |
| chrZ_41525 | 0.928 | UGAGACGGAGACCUCUCUGGUGAGGUGCAGAGCUUAGCUGAUUGGUGAACAGUGAUUGUUUCCCUCUUUGUUCACAGUGGCUAAGUUCUGCACCUGAAGAGAAGGUGA | ..........((((((((....((((((((((((((((((....((((((((.((.........)).))))))))..))))))))))))))))))..)))).)))).. |
| chr1_2080 | 0.928 | GAAGCUGUGUGCAUCCGGGUUGAGGUAGUAGGUUGUAUGGUUUAGAGUUACACCCUGGGAGUUAACUGUACAACCUUCUAGCUUUCCUUGGAGCACACUUGAGCCAUCGAG | ((.(((((((((.((((((..(((.(((.(((((((((((((..((.(..(.....)..).))))))))))))))).))).)))..))))))))))))...)))..))... |
| chr9_35428 | 0.928 | CGGGGGUGGUGGCGGGACGCUCACGAGCGUGCCGAUGAUUUGGCGGCACAGUGGGCAGUUGUUCCUGUCGGCGGCCCAGCGCUCAAUGCAGCGCCUGCAGAAGGUGUGCCUGCA | ..(((.((.(((((((((((((((..((.((((((....))))))))...))))))....).)))))))).)).))).........((((((((((.....)))))...))))) |
| chr1_485 | 0.916 | CUUGCAGAUCUCAGUAACCCCUUUAGAGGAUGACUGAUUUCUUUUGGUGUUCAGAGUCAAUAAUAUUUUCUAGCACCAUUUGAAAUCGGUUAUAGUGAUUGGGGAAUUG | .................((((..(.....((((((((((((...(((((((.((((...........)))))))))))...))))))))))))....)..))))..... |
| chr26_26882 | 0.916 | CUGAGCCAGAAAAAUGUCUCUUACACAGGCUGACCGAUUUCUCUUGGUGUUCAGAGUCUCAGUUUCUGUCUAGCACCAUUUGAAAUCGGUUAUGAUGUAGGGGGAAAAGCA | ................(((((((((((...(((((((((((...((((((((((((.......)))))...)))))))...))))))))))))).)))))))))....... |
| chr9_36157 | 0.914 | UUGCCCUACUUGUUCCGCCCUAGCAGCACGUAAAUAUUGGUGUAGUAAAAUAAACCUUAAACCCCAAUAUUAUUGUGCUGCUUAAGCGUGGCAGAGAUUCAGCAACUUGUU | ((((....(((((..(((...(((((((((..((((((((.((..(((........))).)).))))))))..)))))))))...)))..))).)).....))))...... |
| chr9_36158 | 0.908 | AUUUUUCUGUUAAUGAAUCUGUGCUCCUGACAUGUGGAUGAAUAGAAGAGUUGCCCUACUUGUUCCGCCCUAGCAGCACGUAAAUAUUGGUGUAGUAAAAUAAACCUUAAA | (((((.(((((((((....((((((.(((....(((((.........((((......))))..)))))..))).))))))....))))))..))).))))).......... |
| chr1_8055 | 0.904 | UGCUGAUGUCUGUCAUACUCUAGCAGCACGUAAAUAUUGGUGUUAAAACUGUAAAUAUCUCCAGUAUUAACUGUGCUGCUGAAGUAAGGCUAGCCACUUCUGCAUGUGAGU | (((.((.((..(((.((((.(((((((((((.((((((((.(................).)))))))).)).))))))))).)))).)))..))....)).)))....... |
| chr9_36159 | 0.904 | GUGAGGCCUUAAAGUACUCUAGCAGCACAUCAUGGUUUGCAUGCUGUAGUGAAGAUGCGAAUCAUUAUUUGCUGCUUUAGAAAUUUAAGGAAGAUAAACAGUUGAAGACA | ......(((((((...((..(((((((.((.(((((((((((.((.......))))))))))))).)).)))))))..))...))))))).................... |
| chrUn_NT_465061v1_38058 | 0.9 | ACUGCACACUGGGGCCUACAUUUCCUGAGUGACCUGCAGGGUCUGGCGCUGGGUCAGGGAAUGACCCACAUACCAAGGUCUGGGUGCUCUCAGCUGAUGGCAGGGGUAACCAUCC | .........(((.((((.((((..(((((.(((((...((..((((...(((((((.....)))))))....)).))..)))))).).)))))..))))....))))..)))... |

**Supplemental Table 3.** miRDeep predictions at score level 9 or 10 (46):

| **Chromosomal location** | **miRDeep2 score** | **Precursor sequence** | **Secondary structure** |
| --- | --- | --- | --- |
| chr19_14476 | 2.4e+4 | GGCUGUACCAUCCUGUCGGAUAGCUUAUCAGACUGAUGUUGACUGUUGGAUCUCAUGGCAACAACAGUCGGUAGGCUGUCUGACAUUUUGGUAUCUCUCAUCUGACCGUU | ((..((((((...((((((((((((((((.(((((.(((((.((((........))))))))).)))))))))))))))))))))...))))))..))............ |
| [chr1_2078](../../../../Documents/MobaXterm/slash/RemoteFiles/330478_18_72/pdfs_123456789/chr1_2078.pdf) | 2.1e+4 | CUGUGCUGCCAAUUGGCAUAAACCCGUAGAUCCGAUCUUGUGUUGAAAUGCACUGCACAAGCUCGCUUCUAUGGGUCUGUGUCAGUAUGGUGAUCUGGCAAAAGUUUA | ...(((((((((((((((((.(((((((((..(((.(((((((...........))))))).)))..))))))))).))))))))).)))).....))))..... |
| [chr13_11956](../../../../Documents/MobaXterm/slash/RemoteFiles/330478_18_72/pdfs_123456789/chr13_11956.pdf) | 2.0e+4 | AGCAGCUGGGGGCUCCUCCAUGUCUCCCAGCCCAAGGUGCAGUGCUGCAUCUCUGGUCAAUUGGGAGUCUGAGAUGAAGCACUGUAGCUCGGGAAGGGAGGAACUGUGCCC | .((((.(((((....)))))..((((((..(((.((.(((((((((.((((((.((((......)).)).)))))).))))))))).)).)))..))))))..)))).... |
| [chr6_33562](../../../../Documents/MobaXterm/slash/RemoteFiles/330478_18_72/pdfs_123456789/chr6_33562.pdf) | 1.9e+4 | AUUUGGCUCGUUGUUCCUUUUUCCUAUGCAUAUACUUCUUUGAGAGUUUGAUCUAAAGAGGCAUAGAGCAUGGGAAAAUGGGGCGACUGAGGUACUCCGCCAUUCAUUC | ...((((..((((((((.(((((((((((.(((.(((((((.(((......)))))))))).)))..))))))))))).)))))))).(((...))).))))....... |
| [chr7_34267](../../../../Documents/MobaXterm/slash/RemoteFiles/330478_18_72/pdfs_123456789/chr7_34267.pdf) | 1.8e+4 | CCGAAGAGGCUGGCGCUGGGUUCAAGUAAUCCAGGAUAGGCUGUGGUCUGGCAGUCAGCCUGUUCUAGGUUACUUGGCUCCGGAGCCCGCCCGACGCUUCGCCCUGGAGAUG | .(((((.(((.(((.(((((..(((((((((.(((((((((((..(.....)...))))))))))).)))))))))..))))).))).))).....)))))........... |
| [chr2_17954](../../../../Documents/MobaXterm/slash/RemoteFiles/330478_18_72/pdfs_123456789/chr2_17954.pdf) | 1.8e+4 | UACAGAAGGCUGUCACCUGGUUCAAGUAAUCCAGGAUAGGCUGUAUCCAUUCCUGCUGGCCUAUUCUUGGUUACUUGCACUGGGAGGCAGCCGCAGUGCUGCAAAUGAGG | ..(((..(((((((.((..((.(((((((((.(((((((((((((........))).)))))))))).))))))))).))..)).)))))))......)))......... |
| [chr1_NT_456233v1_random_16290](../../../../Documents/MobaXterm/slash/RemoteFiles/330478_18_72/pdfs_123456789/chr1_NT_456233v1_random_16290.pdf) | 1.4e+4 | GUCUGACUGUCCUUUGGGGUGAGGUAGUAGGUUGUAUAGUUUUAGGGUUAUGCCCUGCCUGUCAGAUAACUAUACAAUCUACUGUCUUUCCUGAAGUGGCUGUGAUAUCAU | (((..((.((((((..(((.(((..((((((((((((((((.(((((.....)))))..........))))))))))))))))..))))))..))).))).)))))..... |
| [chr1_1805](../../../../Documents/MobaXterm/slash/RemoteFiles/330478_18_72/pdfs_123456789/chr1_1805.pdf) | 1.4e+4 | AGUCUGACUGUCCUUUGGGGUGAGGUAGUAGGUUGUAUAGUUUUAGGGUUAUGCCCUGCCUGUCAGAUAACUAUACAAUCUACUGUCUUUCCUGAAGUGGCUGUGAUAUCA | .(((..((.((((((..(((.(((..((((((((((((((((.(((((.....)))))..........))))))))))))))))..))))))..))).))).))))).... |
| [chr12_11210](../../../../Documents/MobaXterm/slash/RemoteFiles/330478_18_72/pdfs_123456789/chr12_11210.pdf) | 1.4e+4 | GAUGCCUGCACUGUGGGAUGAGGUAGUAGGUUGUAUAGUUUUAGGGUCAUACCCGCAACUGGGAGAUAACUAUACAAUCUACUGUCUUUCCUAAAGCAGCAGAAAAUCAAC | (((..((((.((.(((((.(((..((((((((((((((((.....(((...((((....)))).)))))))))))))))))))..)))))))).))..))))...)))... |
| [chr26_26871](../../../../Documents/MobaXterm/slash/RemoteFiles/330478_18_72/pdfs_123456789/chr26_26871.pdf) | 1.4e+4 | UGUACUGCUCUGUGGAGGUGAGGUAGUAGGUUGUAUAGUUUGGUGGGAGGGAUUCUGUCCCAUUUCAGGUGAUAACUAUACAGUCUAUUGCCUUCCUUAAAGAGCAGCAAUA | ....(((((((...((((.((((((((((..(((((((((.(((((((.((...)).))))))).........)))))))))..))))))))))))))..)))))))..... |
| [chr24_26206](../../../../Documents/MobaXterm/slash/RemoteFiles/330478_18_72/pdfs_123456789/chr24_26206.pdf) | 1.4e+4 | UUGACUGCAUGCAUCCAGGUUGAGGUAGUAGGUUGUAUAGUUUAGAAUUACACCAAGGGAGAUAACUGUACAACCUCCUAGCUUUCCUUGGGUCUUGCACAAAGCGGCGUG | ..(.((((.((((((((((..(((.(((.(((((((((((((.....(..(.....)..)...))))))))))))).))).)))..))))))...))))....)))))... |
| [chrUn_NT_467319v1_39025](../../../../Documents/MobaXterm/slash/RemoteFiles/330478_18_72/pdfs_123456789/chrUn_NT_467319v1_39025.pdf) | 1.3e+4 | ACCCCGUGCCCCCCCCGGGGUGAGGUAGUAGAUUGUAUAGUUGGGGGCUCACACGCCCGGCUCGGAGAUAACUAUACAGUCUACUGUCUUCCCUGUGGGGGGUGUAUGGCAU | ...(((((((((((((((((.(((..(((((((((((((((((.((((......))))..........)))))))))))))))))..)))))))).)))))).))))))... |
| [chr12_11207](../../../../Documents/MobaXterm/slash/RemoteFiles/330478_18_72/pdfs_123456789/chr12_11207.pdf) | 1.3e+4 | UACAUGCUUCUCUGUCAGAGUGAGGUAGUAGAUUGUAUAGUUGUAGGGUAGUUAUUUUACCCUGUUCAGGAGAUAACUAUACAAUCUAUUGCCUUCCCUGAGGAGUAAAACA | ....((((((((....((.(..(((((((((((((((((((((((((((((.....))))))).........))))))))))))))))))))))..)))))))))))..... |
| [chr2_21250](../../../../Documents/MobaXterm/slash/RemoteFiles/330478_18_72/pdfs_123456789/chr2_21250.pdf) | 1.2e+4 | CCGCGCCCGAGGAGGAUCCGGUCUCCUGAAGCAAAGUUCUGUGACACUCAGACUCUGGUUACGAUAGCAGUCAGUGCACUACAGAACUUUGUCUCCGGGGGCUGCGGCGGCG | ((((..((.....))...((((((((.((..((((((((((((.((((..(((((((.......))).))))))))...))))))))))))..)).))))))))..)))).. |
| [chr23_26080](../../../../Documents/MobaXterm/slash/RemoteFiles/330478_18_72/pdfs_123456789/chr23_26080.pdf) | 1.0e+4 | UUCGGGGCAGUCAUCGCUGCUGUAAACAUCCUUGACUGGAAGCUGUGAGGUGUCAGCGGGGGCUUUCAGUCGGAUGUUUACAGCUGCAGGCUGCUGCAGUCACCUGGGUG | ...(.(((((((...((.(((((((((((((..(((((((((((.(...((....)).).)))))))))))))))))))))))).)).))))))).)...(((....))) |
| [chr4_32121](../../../../Documents/MobaXterm/slash/RemoteFiles/330478_18_72/pdfs_123456789/chr4_32121.pdf) | 1.0e+4 | AUCUUGGCAGUUCCCAGCUCUGAGAACUGAAUUCCAUGGACUGGUUUCAAUUCCAUGCGUUCAGUCCAUGGUAUUCAGUUCUCUAGCUUGGCUGCAUCAAACUUCACAACUG | ......((((..((.((((..(((((((((((.(((((((((((...((......))...))))))))))).))))))))))).)))).))))))................. |
| [chr23_26082](../../../../Documents/MobaXterm/slash/RemoteFiles/330478_18_72/pdfs_123456789/chr23_26082.pdf) | 9.8e+3 | CCCUACCAUGCUGUAGCACGUGUAAACAUCCUACACUCUCAGCUGUGAACUCGAGGUGGCUGGGAGAGGAUUGUUUACGCCUUCUGCCAUGGAGUGAACAUCUGGUGAGCU | .....(((((..((((...(((((((((((((...((((((((..(.........)..)))))))))))).)))))))))...))))))))).((..((.....))..)). |
| [chr3_29472](../../../../Documents/MobaXterm/slash/RemoteFiles/330478_18_72/pdfs_123456789/chr3_29472.pdf) | 9.6e+3 | GAGCUCUGAGUGACAGGUACUGUAAACAUCCUACACUCUCAGCUGUGGAAACUAAGAAAGCUGGGAGAAGGCUGUUUACUCUCCCUGCCUUGGAAAUCAUCUGGAGGGC | ((..((..((.(.((((....(((((((.(((...(((((((((..(....)......))))))))).))).)))))))....)))))))..))..))........... |
| [chr24_26208](../../../../Documents/MobaXterm/slash/RemoteFiles/330478_18_72/pdfs_123456789/chr24_26208.pdf) | 8.3e+3 | GCUCGUUGCGCCCCUCUCAAUCCCUGAGACCCUAACUUGUGAUGUUUAGCUUUUAAAUCCACGGGUUAGGCUCUUGGGAGCUGUGAGUUGUGCUUUGACAUCUUUAUUUU | ..(((..((((..(((.((.((((.((((.(((((((((((..((((((...)))))).))))))))))).))))))))..)).)))..))))..)))............ |
| [chr1_2082](../../../../Documents/MobaXterm/slash/RemoteFiles/330478_18_72/pdfs_123456789/chr1_2082.pdf) | 8.3e+3 | UCAUAUCAGACUUUUCCUAGUCCCUGAGACCCUAACUUGUGAGGUUUUGUAGCAACAAUCACAAGUCAGGCUCUUGGGACCUAGGCGGAGGGGAACCAGCAGCUUUGGAC | .....((...(((..(((((..((..(((.(((.((((((((.(((....))).....)))))))).))).)))..))..)))))..)))..)).((((.....)))).. |
| [chr2_23595](../../../../Documents/MobaXterm/slash/RemoteFiles/330478_18_72/pdfs_123456789/chr2_23595.pdf) | 7.0e+3 | GAUGUAUAGUAGUCUGUUGCUGUAAACAUCCCCGACUGGAAGCUGUAGCAGCUUGAGCUUUCAGUCAGAUGUUUGCUGCACCUGGCUAUACCUGGACAGCAUCAGGAGGAAAAG | ........((((((.(.(((.(((((((((...(((((((((((...........))))))))))).))))))))).))).).)))))).(((((......)))))........ |
| [chr13_11953](../../../../Documents/MobaXterm/slash/RemoteFiles/330478_18_72/pdfs_123456789/chr13_11953.pdf) | 6.9e+3 | GCGGUCGCCGUGCUCUCAGGGUCCAGUUUUCCCAGGAAUCCCUUAGGCGCUACGUUGGGGAUUCCUGGAAAUACUGUUCUUGGGGCCACGGCUCUGCACCUGGAGACAGCCCG | ((((..((((((..(((((((..((((.((.((((((((((((..((((...)))))))))))))))).)).)))).)))))))..)))))).))))..(((....))).... |
| [chr1_7856](../../../../Documents/MobaXterm/slash/RemoteFiles/330478_18_72/pdfs_123456789/chr1_7856.pdf) | 6.8e+3 | UAAAUGUGGAAAACUUGUACCCCUUUCUACACAGGUUGGGAUCAGUUGCAAUGCUGUGCGUUUCUGUGGUAUUGCACUUGUCCCGGCCUGUUGAGGUUGGUGGGGAUAGAG | .............(((...((((..(((..((((((((((((.(((.(((((((..(........)..)))))))))).))))))))))))..))).....))))...))) |
| [chr8_34310](../../../../Documents/MobaXterm/slash/RemoteFiles/330478_18_72/pdfs_123456789/chr8_34310.pdf) | 6.5e+3 | CGGAGAUCCUGCUCCGUCGCCCCAGUGUUCAGACUACCUGUUCAGGACAAUGCUGUUGUACAGUAGUCUGCACAUUGGUUAGACUGGGCAAGGGAAAGCAGCGACAUGGACU | ......((((((((.(((...(((((((.((((((((.(((.(((.......)))....))))))))))).)))))))...))).)))).)))).................. |
| [chr17_13326](../../../../Documents/MobaXterm/slash/RemoteFiles/330478_18_72/pdfs_123456789/chr17_13326.pdf) | 6.5e+3 | CAGAGGCUCCACUCCGUCUGCCCAGUGUUCAGACUACCUGUUCAGGACUACGAGAUUGUACAGUAGUCUGCACAUUGGUUAGGCUGUGCUGGGAUACACCACACACUGCCAG | ....(((........(((((.(((((((.((((((((.(((.(((..........))).))))))))))).))))))).))))).(((.(((......))))))...))).. |
| [chr1_7858](../../../../Documents/MobaXterm/slash/RemoteFiles/330478_18_72/pdfs_123456789/chr1_7858.pdf) | 6.3e+3 | UUUGUUGUUAAAUUGAACACUGUUCUCUGGUUAGUUUUGCAGGUUUGCAUCCAGCUGUAUGAUACUCUGCUGUGCAAAUCCAUGCAAAACUGACUGUGGCAGUGACAAGUCAG | .................(((((((...((((((((((((((((((((((..((((.(((...)))...)))))))))).)).)))))))))))))).)))))))......... |
| [chr4_31648](../../../../Documents/MobaXterm/slash/RemoteFiles/330478_18_72/pdfs_123456789/chr4_31648.pdf) | 6.3e+3 | AAGAUGCCUUCCGGAGAGGUGCUGCUCACAGUCAGUUUUGCAGGUUUGCAUCCCAGCUUGCUAAAAUUGCUGUGCAAAUCCAUGCAAAACUGACUGUGGUGGUGGUGAAUAGC | .....(((((.....)))))((..(.((((((((((((((((((((((((...((((...........)))))))))).)).)))))))))))))))))..)).......... |
| [chr12_11115](../../../../Documents/MobaXterm/slash/RemoteFiles/330478_18_72/pdfs_123456789/chr12_11115.pdf) | 5.8e+3 | CCUCUUGCCUGAUUCCAGGCUGAGGUAGUAGUUUGUACAGUUUGAGGGUCUAUGAUACCACCCGGUACAGGAGAUAACUGUACAGGCCACUGCCUUGCCUGGGACAGAGCUC | ......(((((.((((((((.((((((((.((((((((((((.....((((.((.((((....))))))..)))))))))))))))).))))))))))))))))))).)).. |
| [chr3_29470](../../../../Documents/MobaXterm/slash/RemoteFiles/330478_18_72/pdfs_123456789/chr3_29470.pdf) | 4.5e+3 | UGCUGUUGACAGUGAGCGACUGUAAACAUCCUCGACUGGAAGCUGUGAAGCAGCAGAUGGGGCUUUCAGUCGGAUGUUUGCAGCUGCCAACUGCCACAGACGUCAAGAAA | ..((((.(.((((..(((.((((((((((((..(((((((((((.(............).))))))))))))))))))))))).)))..))))).))))........... |
| [chr27_27144](../../../../Documents/MobaXterm/slash/RemoteFiles/330478_18_72/pdfs_123456789/chr27_27144.pdf) | 4.2e+3 | AUGCAUCUGUCUUCUAUAUGUACCCUGUAGAUCCGAAUUUGUGUAAAGGAAGUUGGGUCACAAAUUCGUAUCUAGGGGAAUAUGUAGUUGACACAAACACUACAGGUCACAA | .......((((..((((((((.((((.(((((.((((((((((...............)))))))))).))))))))).))))))))..))))................... |
| [chr1_7862](../../../../Documents/MobaXterm/slash/RemoteFiles/330478_18_72/pdfs_123456789/chr1_7862.pdf) | 3.8e+3 | GUUCCAGUAGCUUUCUGCUUUGCAGUCUUCUGUUAGUUUUGCAUAGUUGCACUACAGGAAGAAUGUAGUUGUGCAAAUCUAUGCAAAACUGAUGGUGGCCUGUUAUAAUCUA | .....(((((....)))))..((((.((.(((((((((((((((((((((((((((.......))))...))))))..))))))))))))))))).)).))))......... |
| [chr12_10416](../../../../Documents/MobaXterm/slash/RemoteFiles/330478_18_72/pdfs_123456789/chr12_10416.pdf) | 3.3e+3 | CUCUGCAGCGCCGGCACGGGCAACGGAAUCCCAAAAGCAGCUGUCCCCGCAGCGCCCAGCUGCCCUGGGAUUUCGUUACCCGCGCCUCCGCCUCAGCCGCCGCCGCUACGC | ....((.(((.((((.((((.((((((((((((...(((((((..(.......)..)))))))..)))))))))))).)))).((....))....)))).))).))..... |
| [chr7_34091](../../../../Documents/MobaXterm/slash/RemoteFiles/330478_18_72/pdfs_123456789/chr7_34091.pdf) | 2.6e+3 | CAUUAUGGGAUGGCCUGACUUUGAGCUGUUGAAUUCGGGGCCGUAACACUGUCUGAGAGGUUUAUAUUUCUCACAGUGAACCGGUCUCUUUUUCAGCUGCUUCCUGGC | ............(((.......((((.((((((...(((((((...((((((..(((((((....)))))))))))))...)))))))...)))))).))))...))) |
| [chr19_14784](../../../../Documents/MobaXterm/slash/RemoteFiles/330478_18_72/pdfs_123456789/chr19_14784.pdf) | 2.5e+3 | CCUCUGCCCGCUCCUGGCUGCCCGGCAGCAGUUCUUCAGUGGCAAGCUUUAUGUCCUUCUCUAGUAGCUAAAGCUGCCAGUUGAAGAACUGUUGAAUGUAGCCACGUUCAUC | .........(..(.(((((((....(((((((((((((((((((.((((((.((.((.....))..))))))))))))).))))))))))))))...))))))).)..)... |
| [chr1_936](../../../../DOCUME~1/MobaXterm/slash/RemoteFiles/330478_18_72/pdfs_123456789/chr1_936.pdf) | 2.5e+3 | CCGGGUGCGCCAGGCCUGGCUGAGGUAGUAGUUUGUGCUGUUGGUCGGGUUGUGACAUUGCCCGCUGUGGAGAUAACUGCGCAAGCUACUGCCUUGCUAGUGCUGGUGAUG | .......((((((..(((((.(((((((((((((((((.(((((.(((((.........)))))))........))).))))))))))))))))))))))..))))))... |
| [chr2_18747](../../../../Documents/MobaXterm/slash/RemoteFiles/330478_18_72/pdfs_123456789/chr2_18747.pdf) | 2.5e+3 | GGGAGCAGUUCCACAGGAAUGUCCAGCUGGAAGGGGGGCCGUUACACUGUAAGAGAGUGAGUAGCAGGUCUCACAGUGAACCGGUCUCUUUUCCUGCUGUGUCAUGCC | ((((....))))...((.(((.(((((.(((((((((((((...((((((..((((.(........).))))))))))...))))))))))))).)))).).))).)) |
| [chr1_8055](../../../../Documents/MobaXterm/slash/RemoteFiles/330478_18_72/pdfs_123456789/chr1_8055.pdf) | 2.4e+3 | UGCUGAUGUCUGUCAUACUCUAGCAGCACGUAAAUAUUGGUGUUAAAACUGUAAAUAUCUCCAGUAUUAACUGUGCUGCUGAAGUAAGGCUAGCCACUUCUGCAUGUGAGU | (((.((.((..(((.((((.(((((((((((.((((((((.(................).)))))))).)).))))))))).)))).)))..))....)).)))....... |
| [chr4_32054](../../../../Documents/MobaXterm/slash/RemoteFiles/330478_18_72/pdfs_123456789/chr4_32054.pdf) | 2.4e+3 | UUCUCUGGACAUGACUGUAUCUCUGUGCUUUCAGCUUCUUUACAGUGCUGCCUUGUUGCGUUCAUGUCAAGCAGCAUUGUACAGGGCUAUGAAAGAACAGAGGCAUGCUCUU | ......(..((((......(((((((.(((((((((.((.(((((((((((.(((..(((....))))))))))))))))).)))))..)))))).)))))))))))..).. |
| [chrZ_45173](../../../../Documents/MobaXterm/slash/RemoteFiles/330478_18_72/pdfs_123456789/chrZ_45173.pdf) | 2.4e+3 | GAUGUUGGUCUAGUUCUGUGUGGAAGACUAGUGAUUUUGUUGUUUUUAGAUAAUUAAAUUGACAACAAAUCACAGUCUGCCAUAUGGCACAGAUCAUGCCUCUACAGGACAAGU | ((.((((((((.((.((((((((.(((((.(((((((.(((((((((((....)))))..)))))))))))))))))).)))))))).)))))))).)).))............ |
| [chr28_28209](../../../../Documents/MobaXterm/slash/RemoteFiles/330478_18_72/pdfs_123456789/chr28_28209.pdf) | 2.2e+3 | CUCUGUGGUCUGGCUCUGUGUGGAAGACUAGUGAUUUUGUUGUUAUGAUUUAUAAAGGUGACAACAAAUCAUAGCCUGCCAUACAGCACAGAUCUUCACCCUUGCUGGUAGGAA | .(((..((((((...((((((((.((.(((.((((((.((((((((...........))))))))))))))))).)).))))))))..))))))...(((......))).))). |
| [chr33_30699](../../../../Documents/MobaXterm/slash/RemoteFiles/330478_18_72/pdfs_123456789/chr33_30699.pdf) | 2.1e+3 | GGGUGCCCACCCACCCUGGCAGCGCCCGGGCCGAGGUUCUGUCCUACACUCCGGCUGUGGCUAUGGGCAGUCAGUGCAUCACAGAACUUGGUCCCGGGAGCUCAGCAG | (((((......)))))..(((((.(((((((((((.((((((....((((..((((((........))))))))))....))))))))))).)))))).)))..)).. |
| [chr2_23593](../../../../Documents/MobaXterm/slash/RemoteFiles/330478_18_72/pdfs_123456789/chr2_23593.pdf) | 2.1e+3 | UUAAGCUAACUUUUAGUUCCUGUAAACAUCCUACACUCAGCUAUAACAAGUGGUAGGGCUGGGGGGUGGAUGUUUACUUCAACUGACUUGGAAAGGCAGCUUUCCUGAAUGA | ............((((((...((((((((((.((.(((((((...((.....))..)))))))..))))))))))))...))))))...((((((....))))))....... |
| [chr5_32451](../../../../Documents/MobaXterm/slash/RemoteFiles/330478_18_72/pdfs_123456789/chr5_32451.pdf) | 1.9e+3 | CCCGCCCCGCGCCUCCUCAGCCUCCUUGGUGCAGUGGUUCUUAACAGUUCAACAGUUCUCUAUCAUAAUUGUGAAAUGUUUAGGACCACUUGACCAGCGAGGCCCGGGCAUC | ...((((.(.(((((....((.....((((..(((((((((.((((.((((.(((((.........))))))))).)))).)))))))))..)))))))))))).))))... |
| [chrZ_41525](../../../../Documents/MobaXterm/slash/RemoteFiles/330478_18_72/pdfs_123456789/chrZ_41525.pdf) | 1.7e+3 | UGAGACGGAGACCUCUCUGGUGAGGUGCAGAGCUUAGCUGAUUGGUGAACAGUGAUUGUUUCCCUCUUUGUUCACAGUGGCUAAGUUCUGCACCUGAAGAGAAGGUGA | ..........((((((((....((((((((((((((((((....((((((((.((.........)).))))))))..))))))))))))))))))..)))).)))).. |
| [chrZ_47473](../../../../Documents/MobaXterm/slash/RemoteFiles/330478_18_72/pdfs_123456789/chrZ_47473.pdf) | 1.5e+3 | UUCAUGCAGAGCUGGAGGGGAGGCAAGAUGUUGGCAUAGCUGUUAACCUAAAAACCUGCUAUGCCAACAUAUUGUCAUCUUUCCUGUCUGUUUGCGUCAGAGACAGAUUCUAA | .......(((...((((((..(((((.(((((((((((((.(((........)))..))))))))))))).)))))..)))))).((((((((.(....)))))))))))).. |
| [chr20_24659](../../../../Documents/MobaXterm/slash/RemoteFiles/330478_18_72/pdfs_123456789/chr20_24659.pdf) | 1.4e+3 | CACCUUUGAGGGAGCGGCAGUUAAGACUUGUAGUGAUGUUUAGAUAAUGUAUUACAUGAACAUCACUUUAAGUCUGUGCUACUUCUCUCCUCAUUCUUGUCGGCGGGAAGG | ..((..((((((((.((.(((..(((((((.(((((((((((..(((....)))..))))))))))).)))))))..))).)).))).))))).(((((....))))).)) |

**Supplemental Table 4.** miPIE unique predictions above 90% threshold (27 of 71):

| **Chromosomal location** | **miPIE score** |
| --- | --- |
| chrUn_NT_476229v1_39857 | 1 |
| chrUn_NT_470375v1_39465 | 1 |
| chrUn_NT_467319v1_39026 | 0.994 |
| chr12_11208 | 0.992 |
| chr26_26872 | 0.988 |
| chr24_26204 | 0.988 |
| chr4_31646 | 0.984 |
| chrZ_43419 | 0.972 |
| chr8_34624 | 0.97 |
| chrZ_40851 | 0.97 |
| chr17_13325 | 0.964 |
| chr1_7859 | 0.958 |
| chrZ_41523 | 0.948 |
| chr13_11753 | 0.94 |
| chr4_31644 | 0.94 |
| chr10_9089 | 0.936 |
| chr2_23337 | 0.934 |
| chr2_19127 | 0.932 |
| chr1_7865 | 0.93 |
| chr1_2080 | 0.928 |
| chr9_35428 | 0.928 |
| chr1_485 | 0.916 |
| chr26_26882 | 0.916 |
| chr9_36157 | 0.914 |
| chr9_36158 | 0.908 |
| chr9_36159 | 0.904 |
| chrUn_NT_465061v1_38058 | 0.9 |

**Supplemental Table 5.** miRDeep2 unique predictions above 90% threshold (2 of 46):

| **Chromosomal location** | **miRDeep2 score** |
| --- | --- |
| chr5_32451 | 1.9e+3 |
| chrZ_47473 | 1.5e+3 |

**Supplemental Table 6.** Novel sites predicted above decision threshold by both miRDeep2 and miPIE (44):

| **Chromosomal location** | **miPIE score** | **miRDeep2 score** |
| --- | --- | --- |
| chr7_34267 | 1 | 1.8e+4 |
| chr2_17954 | 0.998 | 1.8e+4 |
| chr6_33562 | 0.998 | 1.9e+4 |
| chr13_11956 | 0.996 | 2.0e+4 |
| chr2_21250 | 0.996 | 1.2e+4 |
| chr19_14476 | 0.996 | 2.4e+4 |
| chr1_2078 | 0.996 | 2.1e+4 |
| chrUn_NT_467319v1_39025 | 0.994 | 1.3e+4 |
| chr12_11207 | 0.992 | 1.3e+4 |
| chr24_26206 | 0.99 | 1.4e+4 |
| chr12_11210 | 0.99 | 1.4e+4 |
| chr26_26871 | 0.99 | 1.4e+4 |
| chr1_1805 | 0.988 | 1.4e+4 |
| chr1_NT_456233v1_random_16290 | 0.988 | 1.4e+4 |
| chr23_26080 | 0.988 | 1.0e+4 |
| chr4_32121 | 0.988 | 1.0e+4 |
| chr23_26082 | 0.988 | 9.8e+3 |
| chr3_29472 | 0.984 | 9.6e+3 |
| chr1_7856 | 0.984 | 6.8e+3 |
| chr12_11115 | 0.984 | 5.8e+3 |
| chr4_31648 | 0.982 | 6.3e+3 |
| chr1_7858 | 0.98 | 6.3e+3 |
| chr1_2082 | 0.978 | 8.3e+3 |
| chr24_26208 | 0.976 | 8.3e+3 |
| chr2_23595 | 0.974 | 7.0e+3 |
| chr13_11953 | 0.972 | 6.9e+3 |
| chr1_7862 | 0.968 | 3.8e+3 |
| chr27_27144 | 0.968 | 4.2e+3 |
| chr8_34310 | 0.964 | 6.5e+3 |
| chr17_13326 | 0.962 | 6.5e+3 |
| chr3_29470 | 0.96 | 4.5e+3 |
| chr19_14784 | 0.96 | 2.5e+3 |
| chr12_10416 | 0.96 | 3.3e+3 |
| chr1_936 | 0.95 | 2.5e+3 |
| chr2_23593 | 0.95 | 2.1e+3 |
| chr33_30699 | 0.948 | 2.1e+3 |
| chr2_18747 | 0.946 | 2.5e+3 |
| chr7_34091 | 0.946 | 2.6e+3 |
| chr20_24659 | 0.944 | 1.4e+3 |
| chr4_32054 | 0.942 | 2.4e+3 |
| chr28_28209 | 0.938 | 2.2e+3 |
| chrZ_45173 | 0.936 | 2.4e+3 |
| chrZ_41525 | 0.928 | 1.7e+3 |
| chr1_8055 | 0.904 | 2.4e+3 |

Supplemental Table 7. Novel microRNA predictions by miPIE at different precision thresholds on the 6 test species

|  | All microRNA predictions | Pr > 80% | Pr > 90% | Pr > 95% |
| --- | --- | --- | --- | --- |
| Chicken | 186 | 106 | 71 | 47 |
| Horse | 400 | 265 | 192 | 143 |
| Cow | 331 | 147 | 105 | 74 |
| Mouse | 382 | 252 | 198 | 150 |
| Human | 181 | 81 | 54 | 43 |
| Fruit-fly | 98 | 50 | 36 | 24 |
